# Crosstalk of necroptosis and pyroptosis defines tumor microenvironment characterization and predicts prognosis in clear cell renal carcinoma

**DOI:** 10.3389/fimmu.2022.1021935

**Published:** 2022-09-30

**Authors:** Liangmin Fu, Jiahao Bao, Jinhui Li, Qiuyang Li, Hansen Lin, Yayun Zhou, Jiangbo Li, Yixuan Yan, Marvin E. Langston, Tianhao Sun, Songliang Guo, Xinwei Zhou, Yuhang Chen, Yujun Liu, Yiqi Zhao, Jun Lu, Yong Huang, Wei Chen, Benjamin I. Chung, Junhang Luo

**Affiliations:** ^1^ Department of Urology, The First Affiliated Hospital of Sun Yat-sen University, Guangzhou, China; ^2^ Institute of Precision Medicine, The First Affiliated Hospital of Sun Yat-sen University, Guangzhou, China; ^3^ Hospital of Stomatology, Guanghua School of Stomatology, Sun Yat-sen University, Guangdong Provincial Key Laboratory of Stomatology, Guangzhou, China; ^4^ Department of Urology, Stanford University Medical Center, Stanford, CA, United States; ^5^ Department of Obstetrics & Gynecology, Third Affiliated Hospital, Sun Yat-sen University, Guangzhou, China; ^6^ Zhongshan School of Medicine, Sun Yat-sen University, Guangzhou, China; ^7^ Department of Epidemiology and Population Health, Stanford University School of Medicine, Stanford, CA, United States; ^8^ Shenzhen Key Laboratory for Innovative Technology in Orthopaedic Trauma, Guangdong Engineering Technology Research Center for Orthopaedic Trauma Repair, Department of Orthopaedics and Traumatology, The University of Hong Kong-Shenzhen Hospital, Shenzhen, China; ^9^ Department of Emergency, The First Affiliated Hospital of Sun Yat-sen University, Guangzhou, China

**Keywords:** clear cell renal cell carcinoma, necroptosis, pyroptosis, tumor microenvironment, prognosis

## Abstract

Pyroptosis and necroptosis are two recently identified forms of immunogenic cell death in the tumor microenvironment (TME), indicating a crucial involvement in tumor metastasis. However, the characteristics of necroptosis and pyroptosis that define tumor microenvironment and prognosis in ccRCC patients remain unknown. We systematically investigated the transcriptional variation and expression patterns of Necroptosis and Pyroptosis related genes (NPRGs). After screening the necroptosis-pyroptosis clusters, the potential functional annotation for clusters was explored by GSVA enrichment analysis. The Necroptosis-Pyroptosis Genes (NPG) scores were used for the prognosis model construction and validation. Then, the correlations of NPG score with clinical features, cancer stem cell (CSC) index, tumor mutation burden (TMB), TME, and Immune Checkpoint Genes (ICGs) were also individually explored to evaluate the prognosis predictive values in ccRCC. Microarray screenings identified 27 upregulated and 1 downregulated NPRGs. Ten overall survival associated NPRGs were filtered to construct the NPG prognostic model indicating a better prognostic signature for ccRCC patients with lower NPG scores (P< 0.001), which was verified using the external cohort. Univariate and multivariate analyses along with Kaplan-Meier survival analysis demonstrated that NPG score prognostic model could be applied as an independent prognostic factor, and AUC values of nomogram from 1- to 5- year overall survival with good agreement in calibration plots suggested that the proposed prognostic signature possessed good predictive capabilities in ccRCC. A high-/sNPG score is proven to be connected with tumor growth and immune-related biological processes, according to enriched GO, KEGG, and GSEA analyses. Comparing patients with a high-NPG score to those with a low-NPG score revealed significant differences in clinical characteristics, growth and recurrence of malignancies (CSC index), TME cell infiltration, and immunotherapeutic response (P< 0.005), potentially making the NPG score multifunctional in the clinical therapeutic setting. Furthermore, AIM2, CASP4, GSDMB, NOD2, and RBCK1 were also found to be highly expressed in ccRCC cell lines and tumor tissues, and GASP4 and GSDMB promote ccRCC cells’ proliferation, migration, and invasion. This study firstly suggests that targeting the NPG score feature for TME characterization may lend novel insights into its clinical applications in the prognostic prediction of ccRCC.

## Introduction

Renal cell carcinoma (RCC) is one of the most common malignancies of the urinary system, and it is also the most common type of kidney cancer in adults. Based on the GLOBOCAN estimates of cancer incidence and mortality produced by the International Agency for Research on Cancer (IARC), an estimated 431,288 new cases of RCC were diagnosed and 179,368 deaths were recorded worldwide in 2020 (1). Additionally, the mortality rate of RCC is decreasing in the majority of developed countries while not in the less-developed regions where access to optimal therapies is still constrained (2). In the United States, the American Cancer Society estimates that 79,000 new cases and 13,920 deaths were estimated in 2022 (3), but the mortality rate from kidney cancer decreased by 2.5% per year between 2015 to 2019 (4). In China, the estimation of new cases and deaths of kidney cancer were 50,088 and 46,345 respectively (5). According to a recent study based on age-period-cohort analysis, the kidney cancer mortality rate displayed a significant increasing trend, with an increase of 2.85% for men and 1.25% for women (6).

RCC consists of many heterogeneous subtypes, and it is canonically categorized into three major histological subtypes, including clear cell RCC (ccRCC), papillary RCC, and chromophobe RCC (7, 8). Clear cell renal cell carcinoma (ccRCC) is the most prevalent histological subtype that accounts for approximately 75% of all RCC cases (9), which is characterized by malignant epithelial cells, arising from the epithelial cells of renal proximal tubules (10, 11). Usually, patients with early stages of RCC do not have signs or symptoms, and the pathologies and carcinogenesis mechanisms are still unclear (12, 13). Additionally, symptoms are hard to identify until kidney cancer has spread to other parts of the body, usually the lymph nodes, lungs, or long bones (14). Currently, surgical intervention remains the primary treatment for RCC patients, especially in the early stages (15). As one of the most lethal urologic malignancies, it has been reported that over 30% of RCC patients relapsed after surgery, around 20%-30% of RCC patients were diagnosed with metastatic disease, and more than 40% of RCC patients died from it eventually (16, 17). Using Surveillance Epidemiology and End Results (SEER) registry a data source sampled to represent the entire US population, found that the prognosis for patients with advanced and metastatic disease is poor, with only 13.6% 5-year survival (18). Accordingly, the introduction and development of specific RCC treatments have improved patients’ outcomes, and integrating surgery or ablative strategies with targeted therapies has been becoming the optimal adjuvant therapy for patients with metastatic RCC (19). In order to ensure appropriate treatment selection for patients, it is necessary to develop a signature to accurately predict the overall survival of ccRCC patients.

Considering that RCC is also an immunogenetic tumor that contains many immune cells such as tumor-infiltrating lymphocytes (TIL) and tumor-infiltrating immune cells (TIICs) (20–22), an alternative to antiangiogenic therapy is targeted as a selection of immunotherapy in RCC patients. Recent findings concluded that treatment with immune checkpoint inhibitors (ICIs) like Nivolumab alone could prolong the overall survival (OS) rate and reduce the grade 3 or 4 adverse events (23). When combined with anti-cytotoxic T-lymphocyte antigen-4 (CTLA4) and anti-programmed death 1 (PD-1) for treatment, the OS and response rates significantly improved (24). Studies have highlighted the importance of tumor microenvironment (TME) to RCC therapy and advocated combination treatment of antiangiogenics and targeted immunotherapy to overcome resistance. It has been recognized as a first-line therapy option currently (25, 26). Pyroptosis and necroptosis are two recently characterized forms of immunogenic cell death (ICD) in the TME, and they are expected to stimulate the immunogenicity of tumors and induce the effectiveness of anti-tumor immune responses (27). Unlike apoptosis, pyroptosis and necroptosis belong to programmed forms of necrosis (28), and they could protect against infections in the TME and be initiated by host and pathogen molecules (29). Currently, the underlying mechanisms of programmed forms of necrosis in RCC are still not fully understood. TME plays an essential role in tumor survival and promotion function in which the tumor cells could disseminate from the primary site to distant locations invasively, which results in cancer metastasis (30), thus, with more understanding of pyroptosis and necroptosis characteristics in the TME and identifying non-apoptotic cell death biomarkers in RCC prognosis predictions could benefit the development of anti-cancer treatment and next-generation chemotherapeutic medicines through targeting at powerful anti-tumor adaptive immune response (31).

In this study, the Gene expression data and clinical information datasets related to ccRCC and healthy control (HC) samples were downloaded from Gene Expression Omnibus (GEO) and The Cancer Genome Atlas (TCGA) databases, aiming to screen the differentially-expressed necroptosis- and pyroptosis- related genes (DE-NPRGs). Then we applied the bioinformatics methods and techniques to get the Necroptosis-Pyroptosis Clusters (NP-Clusters) and constructed the Necroptosis-Pyroptosis Genes (NPG) score for prognosis and functions analysis in ccRCC carcinogenesis and prognosis pathways, which could provide a reliable basis for pathological mechanisms of ccRCC and evidence for the therapeutic targets in clinical treatment and applications.

## Methods

### Acquisition of date and identification of differentially expressed necroptosis-related genes and pyroptosis-related genes (DE-NPRGs)

Gene expression data and clinical information of ccRCC patients were extracted from TCGA database (http://cancergenome.nih.gov/) and the GEO database (https://www.ncbi.nlm.nih.gov/geo/) (32–34). A total of 539 ccRCC samples and 72 normal samples with transcriptional data (HTSeq-FPKM), single nucleotide polymorphism (SNP), copy number variation (CNV), and clinical information were obtained from the TCGA database. The clinical pathological characteristics of these patients are shown in **Table S1** and **Table S2**. The FPKM values of TCGA-KIRC were transformed into transcripts per kilobase million (TPM) for subsequent analysis. In addition, the gene expression profile data and clinical information of the external validation cohort were downloaded from the Gene Expression Omnibus (GEO) (http://www.ncbi.nlm.nih.gov/geo/, ID: GSE29609) (35).

Necroptosis-related genes and pyroptosis-related genes (NPRGs) were extracted following the summary of previous research and reviews (36–40). A total of 60 necroptosis- and pyroptosis- related genes (NPRGs) were obtained and provided in **Table S3**. The “Limma” R package was applied to identify DE-NPRGs between ccRCC samples and normal samples (41). False Discovery Rate (FDR)< 0.05 and |log2 Fold Change| ≥ 1 were regarded as the threshold of differential expression. A protein-protein interaction (PPI) network for the DE-NPRGs was constructed by Search Tool for the Retrieval of Interacting Genes (STRING v11.0, https://string-db.org/) (42).

### Screening of necroptosis-pyroptosis clusters (NP-Clusters) by nonnegative matrix factorization (NMF)

Based on the expression of DE-NPRGs, the NMF algorithm was employed to perform unsupervised clustering using the “NMF” R package (43). K-value was determined when the magnitude of the cophenetic correlation coefficient began to decrease. Next, principal component analysis (PCA) was performed to show the classification of common responsive genes (CRG) clusters. We then investigated the correlation between NP-Clusters with the clinical characteristics and prognosis. The differences in overall survival (OS) between different NP-Clusters were determined with Kaplan–Meier analysis obtained by the “survival” and “survminer” R packages. To investigate the differences of NPRGs in biological functions, gene set variation analysis (GSVA) was conducted with the gene set “c2.cp.kegg.v7.2” obtained from the MSigDB database (https://www.gsea-msigdb.org/gsea/msigdb/) (44, 45). An adjusted P value less than 0.05 was considered statistical significance. Then, single-sample gene-set enrichment analysis (ssGSEA) algorithm was used to quantify the status of immune cell infiltration in ccRCC TME (46).

### Construction and validation of NPG prognostic model

DE-NPRGs were subjected to univariate Cox regression analysis to extract the genes that were associated with OS. First, we excluded the genes with adjusted *P* values over 0.001. Then, the least absolute shrinkage and selection operator (LASSO) Cox regression analysis was performed to avoid the overfitting problem and construct the NPG score signature using the R package “glmnet” (47). The penalty parameter (λ) was selected by applying 10-fold cross-validation according to the minimum criteria. Next, we calculated the NPG score for each sample using the following formula: $NPG\_score=\sum_{i=1}^{n}Coef_{i}\times Exp_{i}$ , with Coef indicating the coefficient and Exp referring to the expression level of each NPRG. The prognostic scoring system for ccRCC patients was established, and the median value of the predicted NPG scores was regarded as the cut-off. PCA and t-distributed stochastic neighbor embedding (t-SNE) analyses were conducted by “stats” and “Rtsne” R packages. The R package “survival” and “survminer” was applied to compare the survival probability between the two groups *via* Kaplan-Meier (K-M) analysis. The R package “timeROC” was employed to perform 1-, 3- and 5- year receiver operating characteristic (ROC) analysis and calculate the value of area under curve (AUC). The external validation GEO cohort was employed to verify the NPG score signature. Patients in the GEO cohort were also divided into high- and low-risk groups. The K-M plot and time-dependent ROC plot were also made.

### Functional annotation of the DEGs between high- and low-risk groups

After dividing patients into high-risk and low-risk groups, we applied the criteria of FDR<0.05 and |log2 Fold Change| ≥1 to screen the DEGs between high- and low-risk groups using the “Limma” R package. On the basis of these DEGs, Gene Ontology (GO) and Kyoto Encyclopedia of Genes and Genomes (KEGG) analyses were carried out with the “clusterProfiler” package (48–50). Gene Set Enrichment Analysis (GSEA) was performed by “clusterProfiler” R package to determine whether prior defined functional or pathway sets of genes differ significantly between high- and low-risk groups (51). The annotated gene sets “h.all.v7.2.symbols” and “c5.bp.v7.2.symbols” from the MSigDB database were adopted in our analysis. Enrichments of gene sets with an adjusted *P* value less than 0.05 were regarded to be significant.

### Independent prognostic analysis and establishment of a nomogram

We extracted the clinical characteristics, including age, pathological stage, and sex of ccRCC patients in the TCGA cohort. These variables, in combination with the NPG score, were analyzed in univariate and multivariable Cox regression analysis. “survival” and “forest” R packages were used for analysis and visualization. In order to provide an applicable tool for clinicians and patients, we establish a nomogram by applying “rms” R package. Age, sex, pathological stage, and NPG score were involved. Time-dependent ROC analysis for survival probability to assess the prognostic accuracy and the calibration plots were applied to compare nomogram-predicated probability with observed outcomes. The clinical usefulness of the nomograms was evaluated by decision curve analysis (DCA).

### Correlation of NPG score with clinical features, cancer stem cell (CSC) index, and tumor mutation burden (TMB)

We conducted a stratified analysis to explore whether the NPG score signature retained its predictive value according to pathological stage I to II and III to IV. Kaplan-Meier analysis (K-M analysis) was applied to compare the difference between high- and low-risk groups. Furthermore, the RNAss file named “StemnessScores_RNAexp_20170127.2.tsv” was downloaded. The tumor stem cell characteristics were obtained from the transcriptome and epigenetics of the samples and then used to evaluate the stem cell-like features of tumors. We performed a correlation analysis to investigate the association between NPG score and Cancer Stem Cell (CSC) index, mutation status, and TMB. The somatic mutation data were obtained from the TCGA database, and the waterfall plots were made employing by “maftools” R package (52).

### Correlation of NPG score with TME, immune checkpoint genes (ICGs)

Two computational methods, ssGSEA, and Cell-type Identification by Estimating Relative Subsets of RNA Transcripts (CIBERSORT), were chosen for immune deconvolution analyses (53). ssGSEA takes the sample gene expression values as the input and computes an overexpression measure for the given gene list of immune cell type relative to all other genes in the transcriptome. CIBERSORT also takes gene expression values as the input but uses a gene expression signature matrix of particular immune cell types instead to compute the infiltration level of each immune cell type. Additionally, we employed the R package “ESTIMATE” to evaluate the TME score (stromal score, immune score, and estimate score) levels between high- and low- risk groups (54). We also investigated the correlations between high- and low-risk groups of the expression levels of ICGs. The Spearman correlation analysis between NPG score and *PDCD1*/*CTLA4* was performed. Additionally, to analyze the response to immune checkpoint therapy, we downloaded immunophenoscore (IPS) data from The Cancer Immunome Atlas (https://tcia.at/ ) (55, 56).

### Therapeutic response prediction

The immunophenoscore (IPS) based on the expression of major immunocompetence determinants was obtained from The Cancer Immunome Atlas (https://tcia.at/) for predicting the clinical benefits of immunotherapy (57, 58). Four types of IPS, including IPS, IPS-CTLA4 blocker, IPS-PD-1/PD-L1/PD-L2 blocker, IPS-CTLA4, and PD-1/PD-L1/PD-L2 blocker, were calculated from the TCGA-KIRC database. Moreover, we explored differences in the chemotherapeutic effects of drugs in ccRCC patients between high- and low-risk groups. Semi-inhibitory concentration (IC50) of anticancer drugs commonly used to treat ccRCC were calculated using the “pRRophetic” R package (59).

### Cell culture and clinical samples

HK-2, 786-O, 769-P, ACHN, A498, CAKI-1, CAKI-2 and OSRC2 were purchased from Procell(Procell Life Science&Technology Co., Ltd). HK2, A498 were cultured in MEM medium supplemented with 10% fetal bovine serum (FBS), 786-O, 769-P, OSRC2 were grown in RPMI-1640 medium with 10% FBS, ACHN was raised in DMEM medium supplemented with 10% fetal bovine serum (FBS), CAKI-1, CAKI-2 were maintained in McCoy’s 5 A medium with 10% FBS. All cells were cultured in a humidified incubator with 5% CO2 at 37°C. All cell lines were authenticated by the short tandem repeat DNA profiling test and tested negative for mycoplasma contamination.

We selected 20 renal cancer tissues and 20 normal tissues from clinical tissue biopsy in The First Affiliated Hospital of Sun Yat-sen University from 2020 to 2022. All the patients were detected by CT and MRI scans of the body and pathology methods. Before collecting samples, the patients were not treated with drugs or radiotherapy. All the studies involving human participants were reviewed and approved by the Institutional Ethics Committee for Clinical Research and Animal Trials Ethical of the First Affiliated Hospital of Sun Yat-sen University [(2021)144], and the Informed Consent Forms were provided and signed by participated patients. All participants agreed on the use of clinical specimens for medical research. The study methodologies conformed to the standards set by the Declaration of Helsinki. One of our authors had access to information that could identify individual participants during or after data collection. In the end, tissues were obtained from 20 ccRCC patients.

### Plasmid construct and siRNA interference

Recombinant plasmids of overexpressing GSDMB and CASP4 were synthesized and constructed by Tsingke (Tsingke Biotechnology Co., Ltd). Lentiviral packaging plasmids and the negative control plasmids were purchased from GeneCopoeia (Rockville, USA). 293 T cells were transfected with GSDMB and CASP4 overexpressing plasmids using Lipofectamine 2000 (Invitrogen, CA, USA); 48 h after the transfection, supernatants containing overexpressing GSDMB and CASP4 lentivirus were collected to transfect 786-O and 769-P cells. Puromycin (5μg/ml) was used for selecting the stably transfected cell lines, and qPCR was used to quantify the efficiency of overexpressing plasmids. Small interference RNA (siRNA) of GSDMB and CASP4 and corresponding negative control were chemically synthesized by RiboBio (RiboBio Co., Ltd China) for further research. The siRNA target sequence is shown in **Supplementary Table S5**.

### RNA isolation and quantitative real-time PCR (qRT-PCR)

Total RNA was extracted using TRIzol Reagent (Invitrogen, USA) according to the manufacturer’s instructions. NanoDrop was used to detect RNA concentration by A260/A280 ratio. PrimeScript RT reagent kit (EZBioscience, China), and SYBR Green PCR reagent (EZBioscience, China) were used to perform cDNA synthesis and further conduct qRT-PCR according to the manufacturer’s protocol. The reaction was incubated at 95°C for 10 min followed by 40 cycles of 95°C for 15 seconds and 60°C for 1 minute. *GAPDH* was used as an internal control. The primer sequences were exhibited in **Supplementary Table S4**. Data were analyzed using the 2-ΔΔCT relative quantification method.

### Immunohistochemistry (IHC) and western blot (WB)

First, the expression of *AIM2, CASP4, GSDMB*, *NOD2*, and *RBCK1* in ccRCC patients was examined by IHC. Antibodies used for IHC were as follows: *AIM2* (Proteintech: #20590-1-AP)*, CASP4* (Proteintech, #11856-1-AP)*, GSDMB* (Proteintech, #12885-1-AP), *NOD2* (Abcam, # ab31488) and *RBCK1* (Proteintech, #26367-1-AP). Second, Western blotting analysis was performed with the standard protocol. ccRCC cells were lysed with NP-40 lysis buffer, and the protein concentration of each sample was measured using a BCA Protein assay kit (Beyotime, China). Equivalent protein was then separated by 10% Tris-Tricine SDS-PAGE and transferred onto polyvinylidene fluoride (PVDF) membranes. After being blocked in 5% fat-free milk, the PVDF membranes were incubated with primary antibodies overnight at 4°C. Primary antibodies included GAPDH (Proteintech, #60004-I-Ig), H3 (Abcam, #ab1791), *AIM2* (Proteintech: #20590-1-AP), *CASP4* (Proteintech, #11856-1-AP), *GSDMB* (Proteintech, #12885-1-AP), *NOD2* (Abcam, # ab31488) and *RBCK1* (Proteintech, #26367-1-AP). Then membranes were incubated with secondary antibody (HRP-conjugated anti-rabbit IgG, Abcam) at room temperature for 1 hour. Finally, the bands on the membranes were observed with a western blot substrate kit (Tanon, Shanghai, China).

### CCK8 and colony formation assays

For CCK8 assays, a total of 1500 ccRCC cells were seeded per well in the 96-well plate. The freshly prepared CCK-8 detection solution was added to the well and incubated for 2 hours at 37 °C. The OD value was detected with a spectrophotometer reader at 450 nm. For colony formation assays, a total of 1000 cells were seeded per well in a 6-well plate. After being cultured for 2 weeks, the colonies were fixed with 4% paraformaldehyde for 20 min at room temperature and then stained with 0.1% crystal violet. The number of colonies (>50 cells) was counted.

### Transwell assays

Transwell migration assays and matrigel invasion assays were performed using a 24-well transwell chamber (Corning, NY, USA) with or without matrigel (Corning, NY, USA). About 50,000 cells were resuspended in serum-free medium and seeded onto the upper chamber, and the lower chamber was added with 10% FBS-containing medium as the chemo-attractant. The cells that migrated through the membrane or invaded through the matrigel were fixed, stained, and then counted under a light microscope.

### Statistical analysis

All statistical analyses were performed using R software (Version 4.1.1) and GraphPad Prism (Version 9.0.0). To compare variables between two groups, we employed the independent sample t-tests for normally distributed continuous variables and Mann-Whitney U tests for nonnormally distributed continuous variables. One-way ANOVA and Kruskal-Wallis tests were used to perform difference comparisons of three or more groups. The survival analysis was conducted *via* the Kaplan-Meier method, and log-rank tests were employed to identify the significance of differences. Correlation coefficients were evaluated by Spearman analysis. The statistical significance was defined with *P*< 0.05.

## Results

### Identification of candidate NPRGs in ccRCC

**Figure 1:**
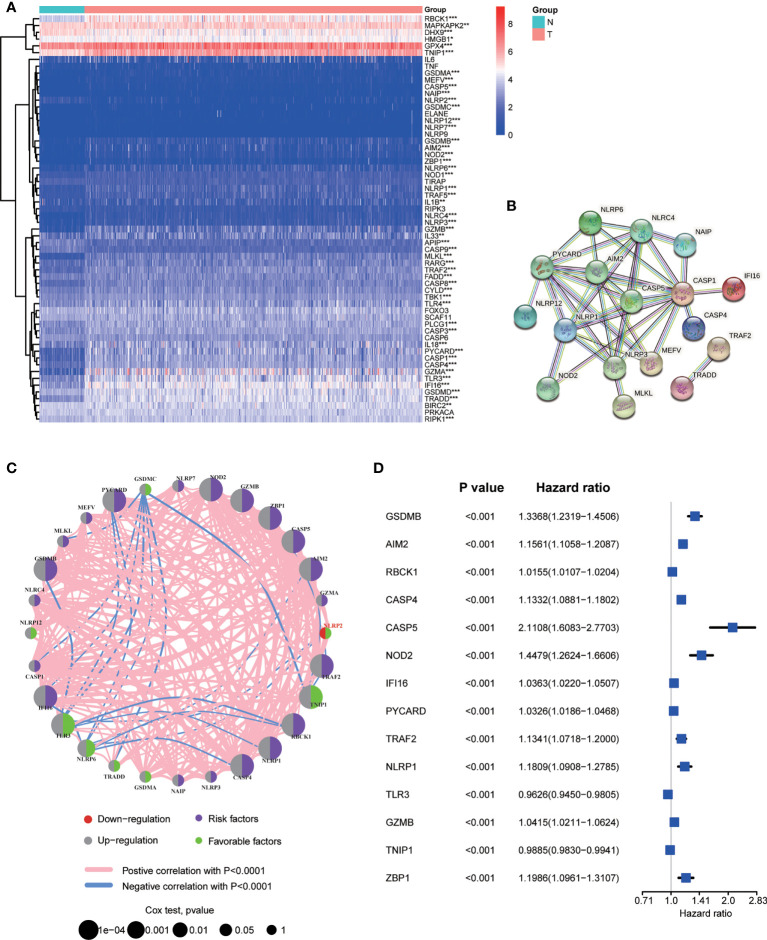
Screening necroptosis and pyroptosis related genes (NPRGs) in ccRCC. **(A)** Heat map of 60 NPRGs. **(B)** PPI network for the cross talks of NPRGs according to STRING database (cross talk score = 0.9). **(C)** The association network landscape of differentially Expressed NPRGs (DE-NPRGs): red dot, down-regulation; grey dot, up-regulation; purple dot, risk factors; green dot, favourable factors; pink line, positive correlation with P < 0.001; blue line, negative correlation with P< 0.0001; the size of dots represents the significance of the correlation. **(D)** Univariate Cox regression analysis of candidate NPRGs with the 14 ones were significantly selected in the model (P < 0.001).

A total of 60 NPRGs were obtained and provided in **Supplementary Table S3**. RNA expression levels of 60 NPRGs between normal and tumor samples were presented in **Figure 1A**. The expression levels of *AIM2, CASP1, CASP4, CASP5, GSDMA, GSDMB, GSDMC, GZMA, GZMB, IFI16, MEFV, MLKL, NAIP, NLRC4, NLRP1, NLRP6, NLRP7, NLRP12, NOD2, PYCARD, RBCK1, TLR3, TNIP1, TRADD, TRAF2, ZBP1* were upregulated in tumor samples, while only the expression level of *NLRP2* was downregulated in tumor samples. The interactions of DE-NPRGs were analyzed by the PPI network according to the STRING database using a confidence of 0.9 as the threshold (**Figure 1B**). The comprehensive landscape of DE-NPRGs interactions, connections, and their prognostic values in ccRCC patients was exhibited in a co-expression network (**Figure 1C**). Then, 28 DE-NPRGs were subjected to univariate Cox regression analysis to select Candidate NPRGs for model construction. A total of 14 NPRGs were retrieved as candidate genes with the adjusted *P* value cutoff of 0.001 (**Figure 1D**).

### Screening of NP-clusters by NMF

**Figure 2:**
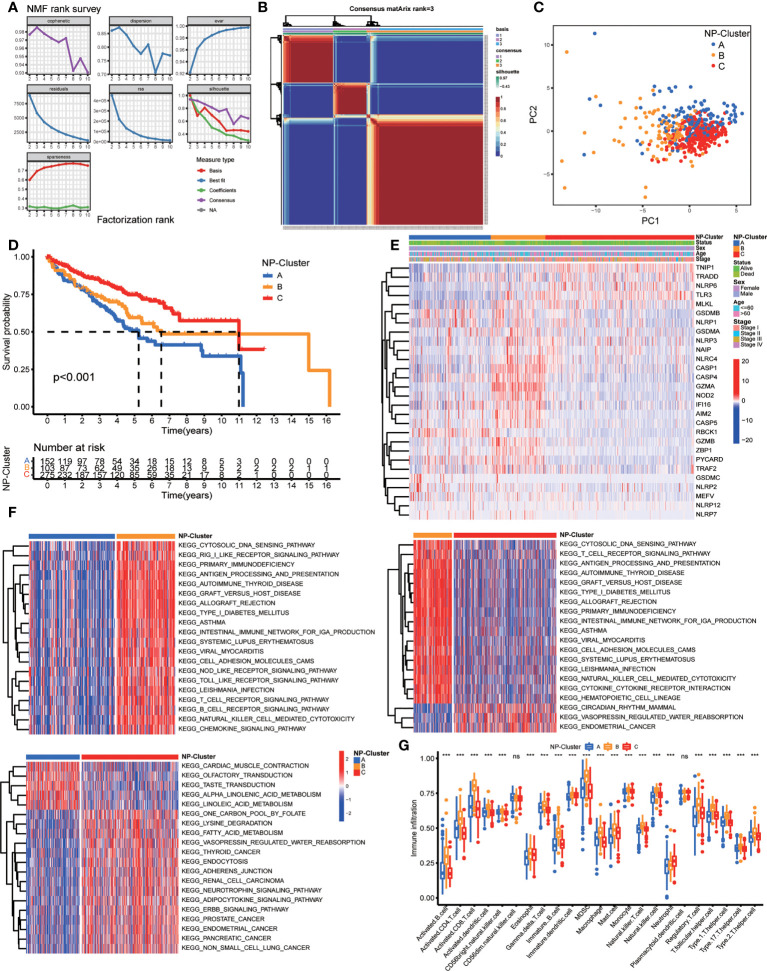
Risk classifications and related functional annotation based on the DE-NPRGs. **(A, B)** Consensus clustering matrix in ccRCC patients with best cluster number of three (k = 3). **(C)** ccRCC patients in TCGA cohort were stratified into three groups. **(D)** Kaplan-Meier curves for the three clusters. **(E)** Heatmap showing the clinicopathologic characteristics formed by DE-NPRGs and clinical features in three NP-clusters. **(F)** Heatmap of GSVA enrichment scores comparisons for the differentially expressed pathways in three NP-clusters. **(G)** Comparison of ssGSEA scores for immune infiltration of ccRCC in three NP-clusters and results visualization. *** P< 0.001; ns, no significance.

In order to explore the expression features and potential biological characteristics of NPRGs in ccRCC, we performed unsupervised clustering analysis to classify patients into distinct NP-Clusters using the NMF algorithm based on the expression levels of 28 DE-NPRGs. The cophenetic correlation coefficient and visual inspection of the consensus matrix suggest the best cluster number was 3 (**Figures 2A, B**, **S1**). Patients with ccRCC in TCGA cohort were categorized into 3 NP-Clusters (cluster A, n=154; cluster B, n=106; cluster C, n=279). PCA was conducted and showed an obvious different distribution among NP-Clusters (**Figure 2C**). Kaplan-Meier OS curves showed that patients in NP-Custer A had the shortest OS time, whereas patients in NP-Cluster C had the superior OS time (**Figure 2D**). **Figure 2E** shows the different expressions of NPRGs and clinicopathological characteristics among NP-Cluster A to C.

To further explore the functional annotation among 3 NP-Clusters, we conducted GSVA enrichment analysis (**Figure 2F**). NP-Cluster B presented enrichment pathways related to immune activation, including the B cell receptor signaling pathway, T cell receptor signaling pathway, NOD like receptor signaling pathways, and Toll like receptor signaling pathways. Next, NP-Cluster A and NP-Cluster C showed an association with immune inhibition compared with NP-Cluster B. NP-Cluster A enriched in nerve conduction related pathways and NP-Cluster C enriched in metabolic-related biological processes. Subsequently, we compared the relevant abundance of immune cells among 3 NP-Clusters to explore the potential function of NPRGs in the immune infiltration of ccRCC by ssGSEA (**Figure 2G**). We observed NP-Cluster B was significantly enriched in immune cell activation, including the higher immune infiltration levels of activated B cells, activated CD4+T cells, activated CD8+T cells, and activated dendritic cells.

### Construction and validation of NPG prognostic model

**Figure 3:**
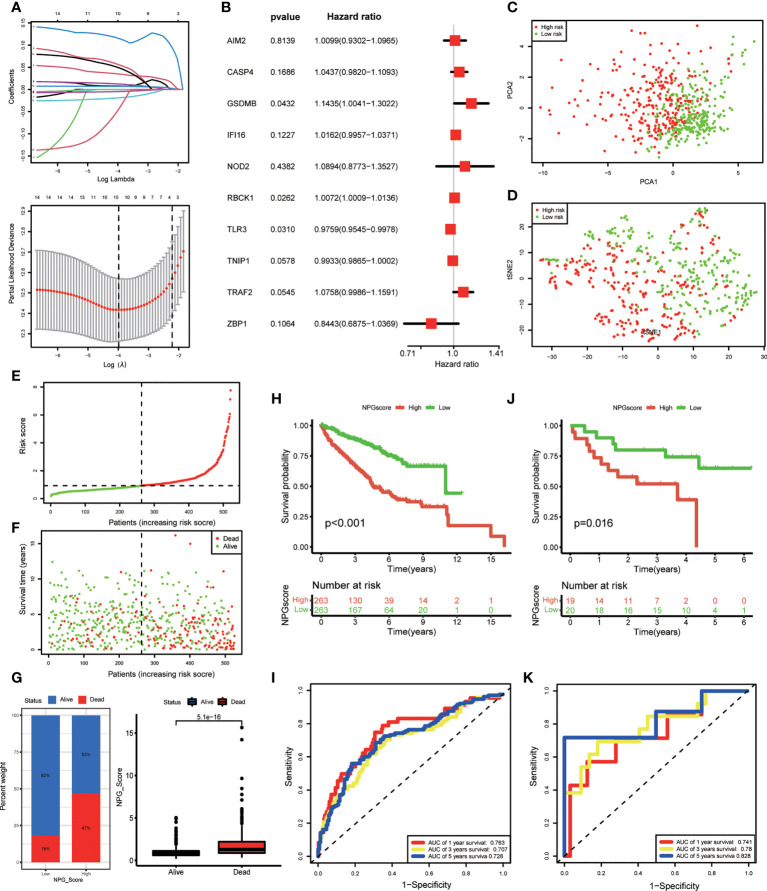
NPG prognostic model construction and validation. **(A)** Cross-verification for fine-tune the selection of parameters in LASSSO regression and 10 NPRGs were obtained in TCGA cohort for the NPG prognostic model construction. **(B)** Multivariate Cox regression analysis of NPRGs for NPG score calculation in TCGA cohort. **(C, D)** PCA map and t-SNE plots for high- and low- risk groups based on the NPG score (red dot, high risk class; green dot, low risk class). **(E, F)** risk plot of NPG score The survival rate (low-NPG score group: on the left side of the dotted line; high-NPG score group: on the right side of the dotted line) and time (red dot, dead subjects; green dot, alive subjects) for each patient in TCGA cohort. **(G)** Correlation of NPG score and vital status of ccRCC patients. **(H)** Kaplan–Meier curves for comparison of NPG score risks between low-NPG score and high-NPG score groups in TCGA cohort. **(I)** ROC curves with the NPG score prediction efficiency in TCGA cohort. **(J)** Kaplan–Meier curves for comparison of NPG score risks between low-NPG score and high-NPG score groups in external validation cohort (GEO cohort). **(K)** ROC curves with the NPG score prediction efficiency in external validation cohort (GEO cohort).

We performed LASSO Cox regression analysis for 14 OS-related DE-NPRGs, and 10 genes were obtained to establish the NPG prognostic model in the TCGA cohort based on the minimum criteria to predict the prognosis of ccRCC patients (**Figure 3A**). The corresponding coefficients were acquired from LASSO Cox regression analysis. NPG score can be calculated as following: NPG_score = (0.009877* AIM2) + (0.042783* CASP4) + (0.134083* GSDMB) + (0.016047* IFI16) + (0.085619* NOD2) + (0.007173* RBCK1) - (0.024375* TLR3) - (0.006708* TNIP1) + (0.073096* TRAF2) - (0.169280* ZBP1). Then, 10 genes were subjected to multivariate Cox regression analysis, and we found GSDMB (HR=1.1435, P<0.05), RBCK1 (HR=1.0072, P<0.05), and TLR3 (HR=0.9759, P<0.05) were independent prognostic factors (**Figure 3B**). Then patients were divided into high-risk (NPG score > median value) and low-risk (NPG score< median value) groups accordingly. PCA and t-SNE plots demonstrated an obvious differential distribution between high and low-risk groups in the TCGA cohort (**Figures 3C, D**). The risk plot of NPG scores revealed that groups with high NPG scores had increased fatal events and shorter survival times (**Figures 3E, F**). Correlation evaluation demonstrated that a low NPG score was linked to a higher percentage of alive patients at follow-up, while a high NPG score was related to a higher percentage of dead patients (**Figure 3G**) at follow-up. In the TCGA cohort, Kaplan–Meier OS curves showed that patients in the high-risk group had a shorter OS than low-risk patients (P< 0.001) (**Figure 3H**). The 1-, 3-, and 5-year survival probability of NPG score were represented by AUC values of 0.763, 0.707, and 0.728, respectively (**Figure 3I**).

To further verify the performance of the prognostic model, we applied an external validation cohort. Similarly, we calculated the NPG score for each sample and divided them into high- and low-risk groups. Kaplan–Meier analysis also showed a significantly better prognosis in the low-risk group compared to that in the high-risk group (**Figure 3J**). ROC curves also verified the accuracy of our signature. As shown in **Figure 3K**, the AUC of the GEO cohort indicated a score of 0.741 at 1 year, 0.780 at 3 years, and 0.828 at 5 years.

### Functional annotation of the DEGs between high- and low-risk groups

**Figure 4:**
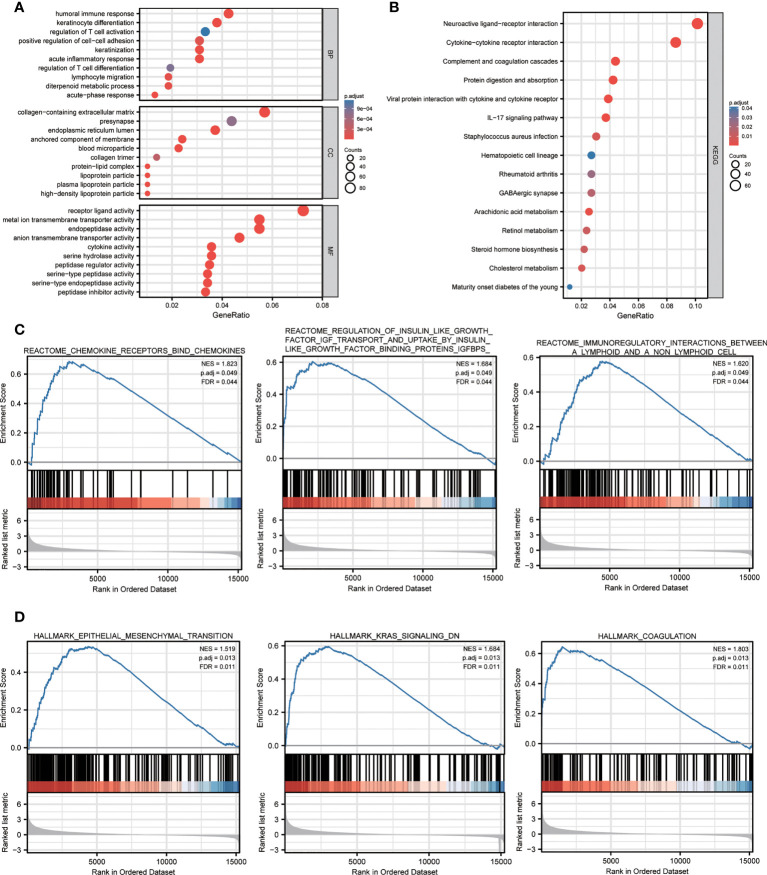
Functional annotation of the DEGs between high- and low- NPG score groups. **(A)** The GO analysis with GO terms of biological processes, cell components, and molecular functions. **(B)** KEGG pathway enrichment analyses of DEGs between high- and low- NPG score groups. **(C, D)** GSEA analysis of tumor progression and immunity between low- and high- NPG score groups.

To elucidate the potential biological functions and signaling pathways associated with high NPG scores, 1536 DEGs between high- and low-risk groups were identified in the TCGA cohort for functional enrichment analysis. The results of GO showed DEGs were enriched in immune-related biological processes (antimicrobial humoral response, humoral immune response, acute inflammatory response, and acute-phase response) and iron-related and signal transport biological processes and molecular functions (organic anion transport, signaling receptor activator activity, and metal ion transmembrane transporter activity) (**Figure 4A**). The results of the KEGG pathway showed that DEGs were associated with pathways including complement and coagulation cascades, cytokine-cytokine receptor interaction, viral protein interaction with cytokine and cytokine receptor, IL-17 signaling pathway, staphylococcus aureus infection, and steroid hormone biosynthesis (**Figure 4B**). Furthermore, GSEA indicated that a high NPG score is predominantly associated with tumor progression and immunity, including (**Figures 4C, D**): reactome chemokine receptors bind chemokines, reactome regulation of insulin like growth factor igf transport and uptake by insulin like growth factor binding proteins igfbps, reactome immunoregulatory interactions between a lymphoid and a non lymphoid cell, hallmark kras signaling dn, hallmark epithelial mesenchymal transition, hallmark coagulation.

### Independent prognostic analysis and establishment of a nomogram

**Figure 5:**
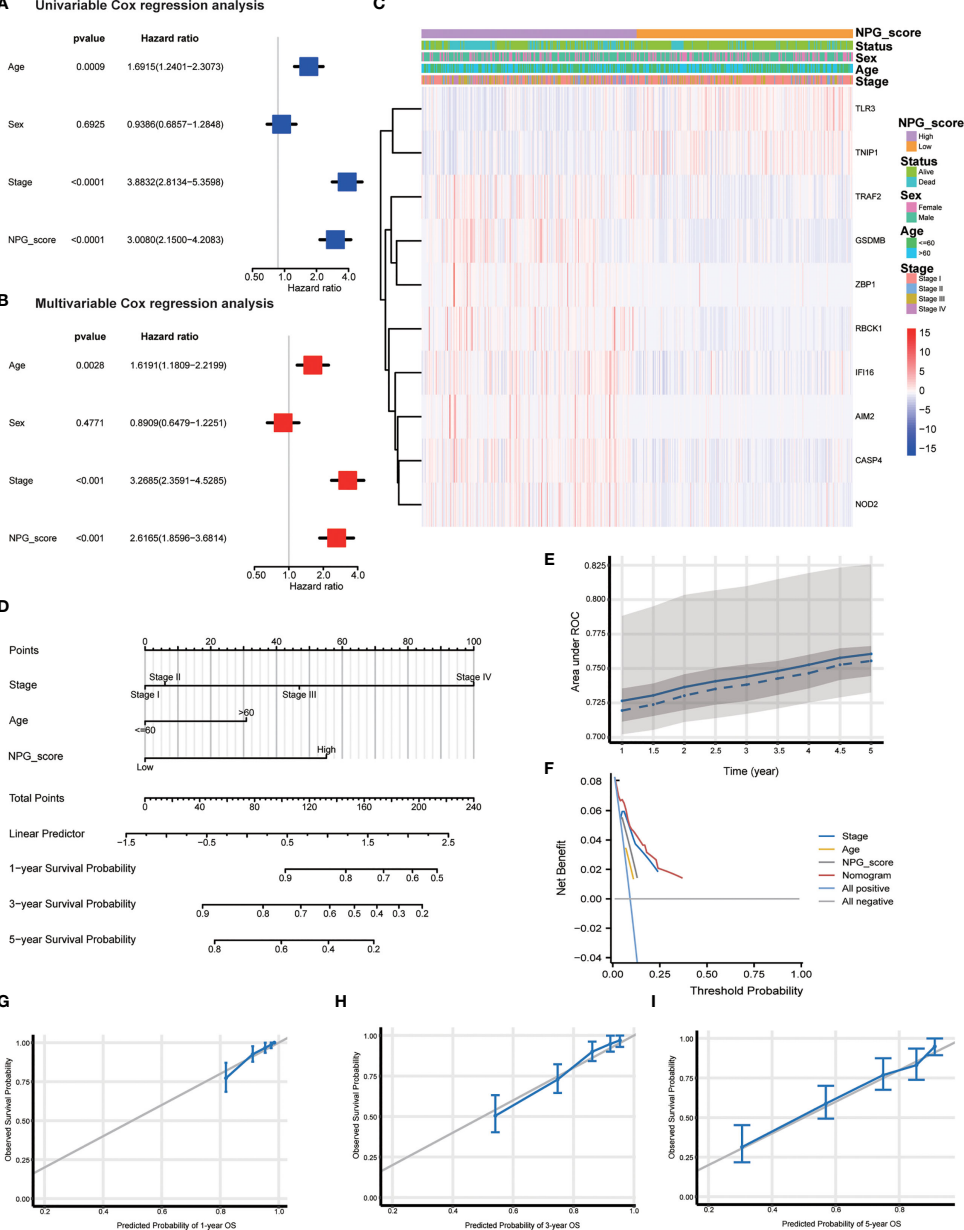
Independent prognostic validation and establish of nomogram. **(A)** Univariate cox regression for TCGA cohort based on the clinical characteristics (pathological stage, age, and sex) and NPG score. **(B)** Multivariate analysis for TCGA cohort. **(C)** Heatmap showing the clinicopathologic characteristics formed by 10 NPRGs and clinical features in low- and high- NPG score groups. **(D)** Nomogram. **(E)** ROC curves illustrating the prediction efficiency of nomogram (AUC, 0.72 to 0.76). **(F)** DCA curves illustrating the clinical effectiveness of the nomogram. **(G–I)** Nomogram to predict 1-, 3-, and 5- year overall survival rates of ccRCC patients. Calibration plots showed overall survival nomogram model to compare the nomogram-predicted probability (blue line) with ideal nomogram (grey line).

To determine whether the NPG score was an independent prognostic predictor for OS, we combined clinical characteristics (pathological stage, age, and sex) and NPG score to perform univariate and multivariate Cox regression analysis. In univariate Cox regression analysis, higher NPG score (HR=3.0080, 95% CI =2.1500−4.2083, P<0.0001), more advanced pathological stage (HR=3.8832, 95% CI =2.8134−5.3598, P<0.0001), and older age (HR=1.6915, 95% CI =1.2401−2.3073, P=0.0009) were significantly related to OS (**Figure 5A**). In multivariate Cox regression analysis, after adjusting for other confounding factors, the NPG score is still confirmed to be an independent predictor for OS (HR=2.6165, 95% CI= 1.8596−3.6814, P<0.001) (**Figure 5B**). Furthermore, comparisons of the clinicopathological characteristics in the high- and low-risk groups revealed significant differences in NPRGs expression and clinicopathological features (**Figure 5C**). High NPG scores were related to advanced pathological stages and more death events. Moreover, the expression of TRAF2, GSDMB, ZBPQ, RBCK1, IFI16, AIM2k, and CASP4 were upregulated in the high-risk group.

To better apply the NPG score prognostic model, we constructed a nomogram based on the TCGA cohort to exhibit a quantitative method to predict the 1-, 3-, and 5-year overall survival. The nomogram contained NPG score and clinical information, including age and pathological stage (**Figure 5D**). AUC values of the nomogram were calculated, and calibration analysis was performed to estimate the predictive ability of the nomogram for prognosis. **Figure 5E** showed the relationship between AUC (0.72 to 0.76) and predicting survival time (from 1 year to 5 years). We plotted DCA curves to illustrate the clinical benefits of the nomogram (**Figure 5F**). The calibration plots showed good agreement between nomogram-predicated probability and the observed outcomes (**Figures 5G–I**).

### Correlation of NPG score with clinical features, CSC index, and TMB

**Figure 6:**
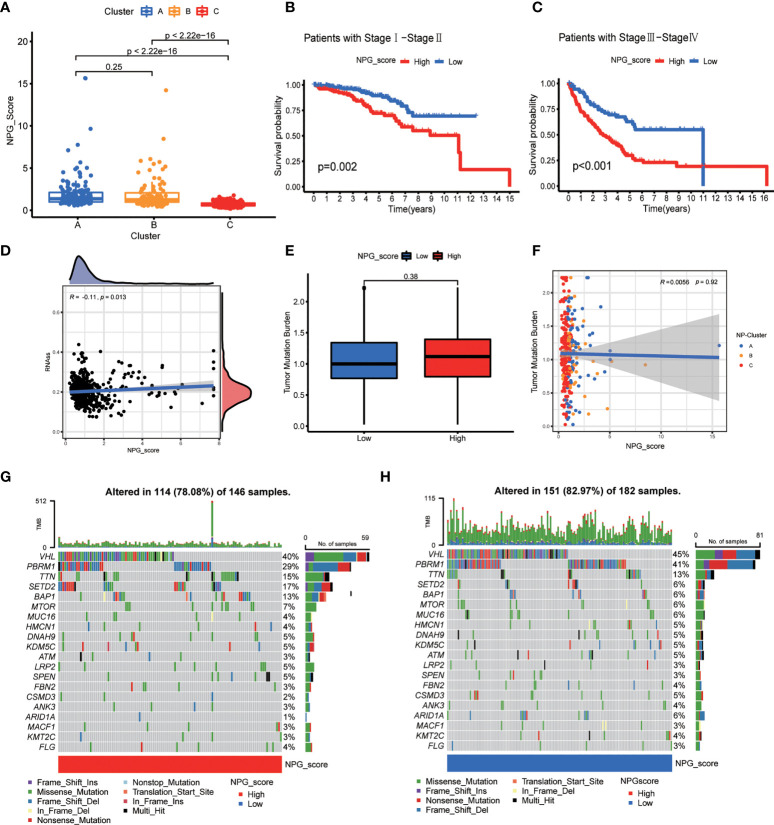
Correlation of NPG score with clinical features, CSC index, and TMB. **(A)** Comparison plot illustrating the differences of NPG score in three NP-Clusters. **(B, C)** Kaplan–Meier curves for comparison of NPG score risks between low-NPG score and high-NPG score groups by stratified analysis of pathological stages. **(D)** Correlation of NPG score and CSC index. **(E, F)** Tumor mutation burden analysis. **(G, H)** Landscape of tumor mutation burden between high- and low- NPG score groups.

We first analyzed the association between NP-Clusters and NPG score, and observed NP-Cluster C had the lowest median NPG score, which was consistent with the K-M analysis of NP-Clusters (**Figure 6A**). However, there was no significance between NP-Cluster A and B (*P*=0.25). Stratified analysis was conducted to evaluate whether the NPG score retained its predictive ability in different pathological stages (stage I–II and stage III–IV). The result showed significantly lower OS in patients in the high-risk group compared to those in patients with low-risk scores for both stage I–II (*P*<0.001) and stage III–IV (*P*<0.001) (**Figures 6B, C**). Previous studies demonstrated that CSC could drive the growth and recurrence of tumors and are resistant to many current treatments (60, 61). A mild but significant negative correlation (*R* = -0.11, *P* = 0.013) was observed between NPG score and CSC index (**Figure 6D**). Furthermore, we described the landscape of somatic mutation between high- and low- risk groups. Patients with high NPG scores had higher mutation frequencies of *TTN*, *SETD2*, *BAP1*, *MTOR*, *LRP2*, *SPEN*, and *FLG*, while *VHL* and *PBRM1* mutation frequencies were much higher in patients with low NPG scores (**Figures 6G, H**). However, there was no significant difference in TMB between high- and low-risk groups (**Figure 6E**). Additionally, Spearman correlation analysis revealed no significant correlation between NP-Clusters and TMB (**Figure 6F**).

### Correlation of NPG score with TME cell infiltration

**Figure 7:**
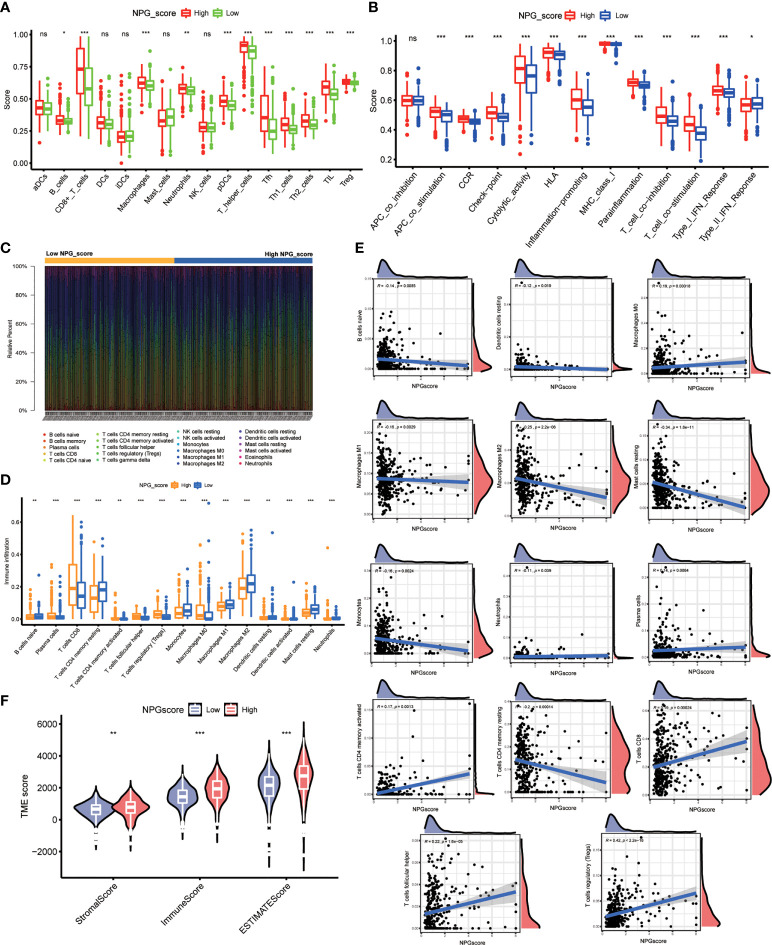
Correlation of NPG score with TME cell infiltration. **(A)** ssGSEA analysis of NPG score and immune infiltration levels in ccRCC. **(B)** Comparison of immune-related functions scores in low- and high- NPG score groups. **(C, D)** Difference in immune cell infiltration levels evaluated by CIBERSORT in ccRCC. **(E)** The relationship of NPG score and different immune cell infiltration levels. **(F)** Correlation analysis between TME scores and NPG score in ccRCC *P < 0.05; ** P < 0.01; *** P< 0.001; ns, no significance.

Then, we employed 2 computational methods, ssGSEA and CIBERSORT, to investigate the correlation between NPG score and TME cell infiltration. Using ssGSEA, we observed the immune infiltration levels of B cells, CD8 T cells, DCs, macrophages, pDCs, T helper cells (*P*<0.001), Tfh, Th1 cells, Th2 cells, TIL, Treg in the high-risk group were significantly higher than those in the low-risk group, while the infiltration levels of mast cells in the high-risk group was lower than that low-risk group (**Figure 7A**). The immune-related functions’ scores include APC co-stimulation, CCR, checkpoint, cytolytic activity, inflammation-promoting, parainflammation, T cell co-inhibition, T cell co-stimulation, Type-I IFN response, and Type-II IFN response were also significantly higher in high-risk group (**Figure 7B**). The difference in immune cell infiltration levels evaluated by CIBERSORT was consistent with ssGSEA (**Figures 7C, D**). Meanwhile, Macrophage M0, Plasma cells, T cells CD4 memory activated, T cells CD8, T cells follicular helper and Tregs were positively correlated with NPG score, while B cells naïve, Dendritic cells resting, Macrophage M1, Macrophage M2, Mast cells activated, Monocytes, Neutrophils, and T cells CD4 memory resting were negatively correlated with NPG score (**Figure 7E**). TME scores, including the Stromal, Immune, and ESTIMATE scores, were significantly higher in the high-risk group (**Figure 7F**).

### Therapeutic response prediction

**Figure 8:**
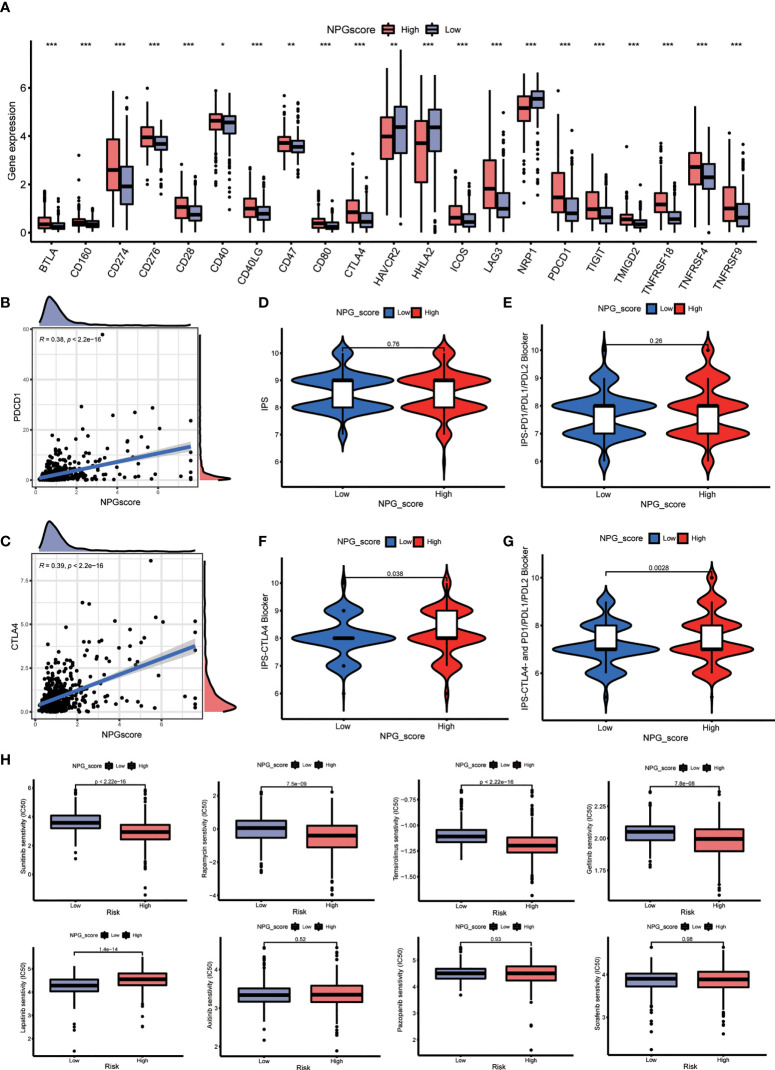
Therapeutic response prediction. **(A)** Comparison the difference of ICGs expression between high- and low-NPG score groups. **(B, C)** The relationship of NPG score and PDCD1 and CTLA4. **(D–G)** Correlation analysis between NPG score and response to immunotherapy. **(H)** Comparison of the sensitivities to the chemotherapy drugs currently used for ccRCC treatment. *P < 0.05; ** P < 0.01; *** P < 0.001.

Considering that the expression levels of ICGs have been reported to associate with the clinical benefit of checkpoint blockade immunotherapy, we compared the difference in ICGs expression between high- and low-risk groups. The expression levels of 19 ICGs containing *BTLA*, *CD274*, *CD276*, *CD40*, *CTLA4*, and *PDCD1* were upregulated in the high-risk group, while *HAVCR2* and *HHLA2* were downregulated (**Figure 8A**). The expression levels of *PDCD1* and *CTLA4* also increased with the increasing NPG score (**Figures 8B, C**). Subsequently, we explored the correlation between NPG score and response to immunotherapy. No significant differences of PD-L1 or PD-L2 expression in the groups of low- NPG and high- NPG score groups (P>0.05 **Figure S2**). However, IPS difference showed that patients with higher NPG scores, who received single *CTLA4* blocker treatment (*P*=0.038) or *CTLA4* and PD-1/PD-L1/PD-L2 combined therapy (*P*=0.0028), could have a better therapeutic effect than those with lower NPG score (**Figures 8D–G**). It revealed that patients with high NPG scores were more suitable for immune checkpoint inhibitor combined therapy. We next obtained chemotherapy drugs currently used for the treatment of ccRCC to estimate the sensitivities of patients in the low- and high-risk groups to these drugs (**Figure 8H**). We observed that the patients in the high-risk group had lower IC50 values for Sunitinib, Rapamycin, and Temsirolimus. The IC50 value of Lapatinib was higher in patients with a high NPG score

### The expression levels of *AIM2*, *CASP4*, *GSDMB*, *NOD2*, and *RBCK1*

**Figure 9:**
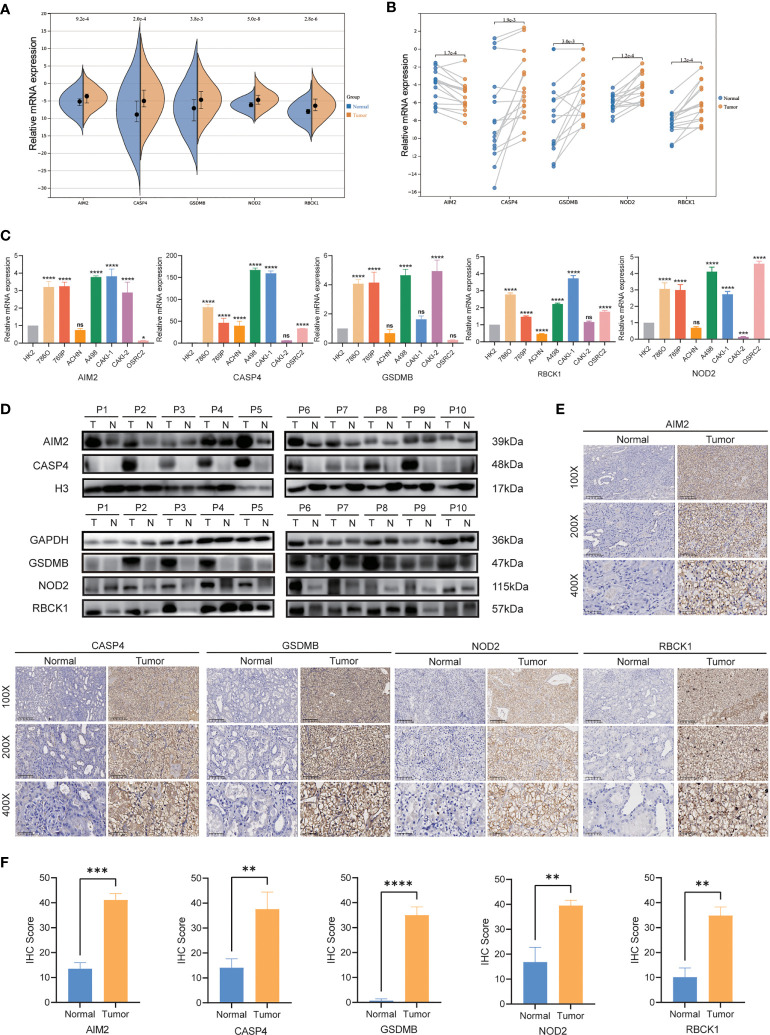
qRT-PCR, IHC and WB verification. **(A, B)** mRNA expression of *AIM2, CASP4, GSDMB, NOD2*, and *RBCK1*in ccRCC patients by qRT-PCR. **(C)** Levels of the mRNA expression in different cell lines as assessed by qRT-PCR analysis. **(D)** Protein expression of *AIM2, CASP4, GSDMB, NOD2*, and *RBCK1* in ccRCC patients by Western blot. **(E, F)** Expression of *AIM2, CASP4, GSDMB, NOD2*, and *RBCK1* makers in ccRCC tumor tissue and normal tissues by IHC. *P < 0.05; ** P < 0.01; *** P< 0.001; **** P< 0.001; ns, no significance.

Then, we investigated the expression levels of *AIM2*, *CASP4*, *GSDMB*, *NOD2*, and *RBCK1* in cell lines and tumor tissues by qRT-PCR, IHC. As shown in **Figures 9A, B**, qRT-PCR showed that the expression of *AIM2*, *CASP4*, *GSDMB*, *NOD2*, and *RBCK1* was significantly upregulated in tumor samples. In our validation results in cell lines, the expression tendency of 5 genes was consistent with prediction results (**Figure 9C**). Additionally, oligo sequences used in quantitative real-time PCR were displayed in **Supplementary Table S4**. IHC staining validated that the protein levels of *AIM2*, *CASP4*, *GSDMB*, *NOD2*, and *RBCK1* in tumor tissues were much higher than that in adjacent normal tissues (**Figure 9D**). To further confirm these findings, their protein expression levels were examined using WB in 10 paired RCC tissues (**Figures 9E, F**). The expression levels of *AIM2*, *CASP4*, *GSDMB*, *NOD2*, and *RBCK1* were frequently higher in RCC tissues.

### *CASP4* and *GSDMB* have promoting effects on the proliferation, migration, and invasion of ccRCC cells

**Figure 10:**
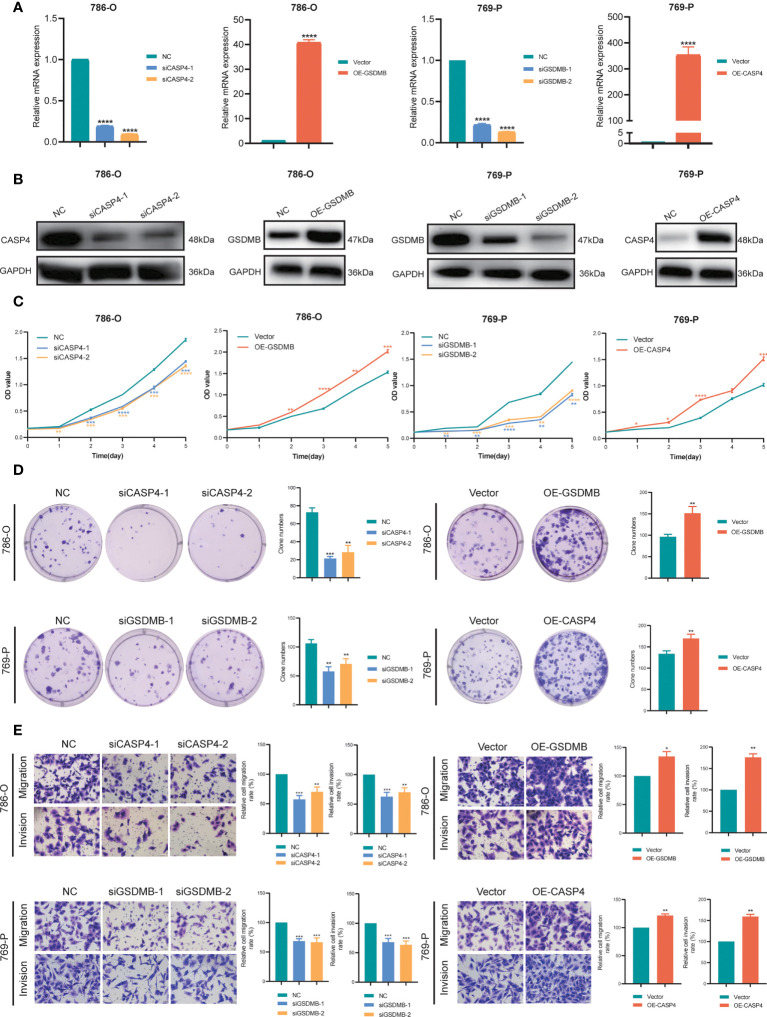
Verification of *CASP4* and *GSDMB* for proliferation, migration, and invasion in ccRCC. **(A, B)** Construction and verification of two siRNA specifically targeting at *CASP4* (siCASP4-1, si-CASP4-2) and *GSDMB* (siGSDMB-1, siGSDMB-2) respectively, and overexpressing vector (OE-CASP4 and OE-GSDMB). **(C, D)** CCK-8 assays, and colony formation assays to detect ccRCC cell proliferation. **(E)** Transwell migration/invasion assay to analyse the ability of ccRCC cell migration and invasion. *P < 0.05; ** P < 0.01; *** P< 0.001; **** P< 0.001.

To explore the role of *CASP4* and *GSDMB* in ccRCC proliferation, migration, and invasion *in vitro*, we constructed two siRNA specifically targeting *CASP4* (siCASP4-1, si-CASP4-2) and *GSDMB* (siGSDMB-1, siGSDMB-2) respectively, and overexpressing vector (OE-CASP4 and OE-GSDMB) (**Figures 10A, B**). CCK-8 assays and colony formation assays showed that silencing *CASP4* and *GSDMB* significantly reduced the proliferative capabilities of ccRCC cells (786-O and 769-P) while overexpressing *CASP4* and *GSDMB* promoted cell proliferation (**Figures 10C, D**). Moreover, transwell assays showed that the migration and invasion abilities of ccRCC cells (786-O and 769-P) were significantly reduced when *CASP4* and *GSDMB* were silenced. In contrast, increasing the expression level of *CASP4* and *GSDMB* increased the migration and invasion rate of cells (**Figure 10E**). In summary, these results collectively confirmed that *CASP4* and *GSDMB* have promoting effects on the proliferation, migration, and invasion of ccRCC cells.

## Discussion

In the present study, from the 60 DE-NPRGs identified as associated with overall survival (OS) in the TCGA cohort., 26 upregulated genes and only one downregulated gene were discovered from the ccRCC samples, compared to the normal kidney samples. Through PPI network analysis based on the STRING database and co-expression network analysis among the DE-NPRGs above, 14 DE-NPRGs were significantly screened as hub genes in the ccRCC group. All of these genes except *TLR3* (HR=0.9626) and *TNIP1* (HR=0.9985) were associated with increased risk with a hazard ratio over 1. To obtain an in-depth understanding of the expression features in the tumor microenvironment and potentially predictable prognosis characteristics for these DE-NPRGs in ccRCC carcinogenesis, three NP-Clusters were identified with different lengths of the OS periods and pathways enrichments in ccRCC. NP-Cluster B was considerably enriched in immune cell activation, including greater numbers of activated B cells, CD4+ T cells, CD8+ T cells, and dendritic cells. Subsequently, the NPG prognostic model was constructed by multivariate Cox regression analysis with 10 genes (*AIM2, CASP4, GSDMB, IFI16, NOD2, RBCK1, TLR3, TNIP1, TRAF2, ZBP1*) that were selected and confirmed with LASSO regression. Then *GSDMB* and *RBCK1* were proved to be harmful prognostic factors, while *TLR3* was expected to be a good prognostic factor independently. This finding is consistent with the report from previous research. Cui et al. found that upregulation of *GSDMB* in ccRCC is associated with immune infiltration and poor prognosis (62); Yu et al. showed that *RBCK1* could indicate the poor prognosis in RCC patients *via* promoting p53 degradation and ubiquitination (63); another recent study from Liao et al. reported *TLR3* could be a promising prognostic biomarker for RCC microenvironment by immune infiltration (64). By the NPG prognostic model we constructed, the TCGA cohort patients were divided into high-risk and low-risk groups, and it is evident that the higher NPG score was significantly associated with worse OS as an independent prognosis predictor in ccRCC patients. The K-M plot of the validation dataset of GEO cohort showed a strongly consistent and comparable result with the TCGA cohort, with AUC of 0.741 at 1 year, 0.780 at 3 years, and 0.828 at 5 years survival, indicating a good prediction of OS by NPG score.

Furthermore, we displayed the calculated NPG score showed 1) significantly positive associations with different pathological stages (stage I–II and stage III–IV); 2) significant but mild correlations with the CSC Index that could promote the growth and recurrence of malignancies of tumors; 3) significant positive correlation with the TME score that contains stromal, immune, and ESTIMATE scores, which was approved by ssGSEA and CIBERSORT consistently; 4) positively suitable to ICIs combined treatment; and the high-NPG score could indicate more sensitive to the chemotherapy drugs of Sunitinib, Rapamycin, and Temsirolimus. This study demonstrated the significance of the NPG score in defining the TME characteristics and predicting the prognosis in ccRCC. It is well known that TME consists of immune and non-immune stromal components related to tumor oncogenesis and malignant behavior (65). Therefore, the abundant immune components in TME could help to estimate immune and stromal cell infiltration, such as ESTIMATE scores (66). Consistently, the NPG score from this study showed that a higher NPG score indicated higher stromal, immune, and ESTIMATE scores in ccRCC, and could be a potentially promising biomarker for ccRCC TME environment evaluation. Moreover, immunotherapy with ICIs has led to cancer therapeutic advancements (67). Given the general tendency of tumors to resist apoptosis or poor response to ICIs, improvement in underlying resistance to apoptotic cell death would be expected to provide the efficiency for immunotherapy. We found that the NPG score could be a potential candidate biomarker for predicting the clinical benefits of immunotherapy. By applying the differences in the chemotherapeutic effects of medications in ccRCC patients between high- and low-risk NPG groups, it is obvious that IC50 of anticancer drugs was lower in the higher NPG score group for Sunitinib, Rapamycin, and Temsirolimus, but not in Lapatinib. According to the previous research, pyroptosis is a microbial ICD produced by immune cell caspases (68); necroptosis is another form of ICD that death receptors (DRs) or pattern recognition receptors (PRRs) recognize adverse cellular environmental to launch necroptosis (67). Thus, we first established the NPG score to engage the extensive crosstalk of pyroptosis and necroptosis which could provide more evidence of multiple cell death modalities to predict the prognosis of ccRCC.

Finally, we utilized experiments *in vitro* to explore the expressions of primary genes of NPG score signature in the carcinogenesis process of ccRCC. Our results illustrated that *AIM2, CASP4, GSDMB, NOD2*, and *RBCK1* had higher expression in RCC tissues, and *CASP4* and *GSDMB* could promote proliferation, migration, and invasion of ccRCC cells. Pyroptosis and necroptosis are two forms of programmed cell death to trigger inflammatory responses with different mechanisms and pathways. Pyroptosis is mediated by members of the Gasdermins family, such as GSDMD and GSDME, to form membrane pores to allow the release of proinflammatory cytokines, IL-1β, and IL-18 (68). Necroptosis is induced by MLKL to also form pores on the cell membrane to release DAMPs. Although triggered by different mechanisms and pathways, they may share a common driver, NLRP3 inflammasome to cause inflammation (68). Additionally, pyroptosis and necroptosis are both found to have crosstalk with antitumor immunity. Pyroptotic cells send danger signals to recruit more CD8+ T cells and other tumor-suppressive cells. Also, induction of pyroptosis strengthens the efficiency of immune checkpoint inhibitors in the “cold tumor”. As for necroptosis, both DAMPs released from necroptotic tumor cells and NF-κB-derived signals released from necroptotic cells can enhance cytotoxic effects by CD8+ T effector cells (40). However, pyroptosis and necroptosis are reported to antagonize the antitumor immune response as well (67). Hence, how to promote anticancer synergy between the two cell deaths is critical to cancer immunotherapy. A recent series of studies displayed the immune landscape of ccRCC and suggested the potential immunotherapeutic targets for ccRCC treatment. A similar finding was observed that assessing the pyroptosis patterns could inform the tumor status and guide immunotherapy strategies by investigating the response of *AIM2* to immunotherapy in ccRCC (69). Also, as in many other types of cancer, *NOD2* was downregulated in ccRCC to promote metastasis (70). Additionally, as an essential for *NF-κB* stimulation, *RBCK1* mutations are shown to be related to immunodeficiency and tumor-infiltrating immune cells, which proved to be an independent prognostic biomarker in RCC (71, 72). Moreover, consistent with our findings, another study by Jiang et al. identified the significance of *CASP4* and *GSDMB* to the immune microenvironment and molecular heterogeneity in ccRCC by a pyroptosis-related prognosis prediction model. Previous studies have shown that *CASP4* protein could involve the activity of cellular processes such as cell inflammation and apoptosis (73); while the *GSDMB* family could manage cell differentiation and proliferation, although the comprehensive role of *GSDMB* has not been fully understood (62). Here, taking the results of the functional enrichment analyses of NPG score-related signaling pathways (tumor progression and immunity) together, it was discovered that the NPG score might be an essential biomarker that influences carcinogenesis (proliferation, migration, and invasion) and prognosis in ccRCC.

Currently, it remains difficult to identify the early symptoms of RCC, and the majority of patients are identified at an advanced stage or even with metastases. In addition, due to the complexity of its etiology and pathophysiology, RCC exhibits the clinical features of a high risk of recurrence and metastasis (74, 75). Although the broader implementation of various diagnostic, screening techniques and advanced therapies, the risk of metastasis and recurrence for ccRCC shows a 5-year survival rate of 50%-69% for ccRCC patients and 10% for patients with metastasis (76). Consequently, it is essential to research the mechanisms underlying the carcinogenesis of ccRCC and to uncover the innovative prospective diagnosis, treatment, and prognosis targets. Advances in cancer bioinformatics analysis could benefit the investigation of genes associated with cancer metabolisms, signaling, communication, and proliferations by combining bioinformatics methodologies (clinical informatics, medical informatics, mathematics, omics science, etc.) (77); thus, it could support in tackling the clinically relevant challenges of early diagnosis, therapy, and prognosis improvement. By adopting the bioinformatics analysis, this study identified 14 OS-related differentially expressed DE-NPRGs, and 10 of them were applied to establish the NPG prognostic model by achieving the NPG score. More importantly, this necroptosis- and pyroptosis- defines tumor microenvironment characterization and prognosis predictive functions were validated by our validation cohort and experiments in ccRCC tissue and cells. However, there are a few limitations that exist still. First, the publicly available online databases were applied for the analysis and validation. It would benefit more ccRCC patients if controlled and multicenter clinical studies and sample examinations could be performed further. In addition, the underlying mechanisms and pathways involved in the pyroptosis-necroptosis genes interactions and their functions for the tumor microenvironment in ccRCC require further exploration. Further experiments *in vivo* and *in vitro* validation would gain more comprehensive knowledge targeting the better application of the NPG model to predict ccRCC prognosis. Also, the multifaceted role of NPRGs and interactions in TME illustrated the necessity to potentially design new NPG score-based immunotherapies to improve the prognosis in ccRCC. Nevertheless, despite the limitations mentioned above, it is undeniable that this study was the first to complete a comprehensive investigation of NPRGs in ccRCC and distinguished and characterized the high- and low- risk groups based on the NPG scores, which might serve as an independent indicator for evaluating prognosis in ccRCC patients. Finally, molecular medicine experiments focusing on immune and prognostic analysis were not enough like IHC for immune cell molecular markers in RCC patients and flow cytometry sorting technology which would be deeply taken into consideration in our further research related to the correlation of NPG with immune activities, allowing us to explore specific changes in the tumor microenvironment.

## Conclusion

This study was the first to offer, to the best of our knowledge, thorough evidence of the substantial interplay between necroptosis-pyroptosis defined tumor environment and prognosis prediction of ccRCC. The NPG score was identified as a potential prognostic biomarker for ccRCC. The NPG score-based dependable and referable risk model accurately predicted the tumor microenvironment and OS of ccRCC. Additional clinical research and biomolecular investigation would be required to provide additional information for future in-depth studies that will profit more ccRCC patients.

## Data availability statement

The original contributions presented in the study are included in the article/**Supplementary Material**. Further inquiries can be directed to the corresponding authors.

## Ethics statement

The studies involving human participants were reviewed and approved by Institutional Ethics Committee for Clinical Research and Animal Trials Ethical of the First Affiliated Hospital of Sun Yat-sen University [(2021)144]. The patients/participants provided their written informed consent to participate in this study.

## Author contributions

LF, JB, JLi, and QL contributed equally to this work. JB, JLi, JBL, QL, SG, YY, YYZ, YL, and YQZ: conceptualization, methodology, software, data curation, formal analysis, and validation. LF, HL, TS, XZ, YC, JLi, JB, JLu, YH, and WC: sample collection, experiments conduction, pathology expertise, data analyzation, interpretation and visualization. JLi and JB drafted and edited the manuscript. ML, BC, and JLuo: manuscript review and edit. BC and JLuo contributed equally to the correspondence work. BC & JLuo: Study coordination, guarantors, writing review. All authors contributed to the article and approved the submitted version.

## Funding

This work was supported by The National Science Fund for Distinguished Young Scholars (No.81725016), and The National Natural Science Foundation of China (No.81872094, No.81772718 and No.82002684), Guangdong Basic and Applied Basic Research Foundation (2020A1515010086).

## Conflict of interest

The authors declare that the research was conducted in the absence of any commercial or financial relationships that could be construed as a potential conflict of interest.

The reviewer HL declared a shared affiliation with the authors JL, ML and BC to the handling editor at the time of review.

## Publisher’s note

All claims expressed in this article are solely those of the authors and do not necessarily represent those of their affiliated organizations, or those of the publisher, the editors and the reviewers. Any product that may be evaluated in this article, or claim that may be made by its manufacturer, is not guaranteed or endorsed by the publisher.

## Supplementary material

Supplementary Figure 1 The visual inspection of the consensus matrix by nonnegative matrix factorization.

Supplementary Figure 2 Difference in PD-L1 and PD-L2 expression levels between high and low NPG score groups.

Supplementary Figure 3 Correlations between the abundance of immune cells and AIM2, CASP4, GSDMB, NOD2, and RBCK1.

Supplementary Figure 4 Functional annotation of CASP4 and GSDMB by GSEA.
