# Supplementary figures and images for: Crosstalk of necroptosis and pyroptosis defines tumor microenvironment characterization and predicts prognosis in clear cell renal carcinoma
Source: Front Immunol. 2022 Sep 30;13:1021935. doi: 10.3389/fimmu.2022.1021935 (PMC9561249; doi:10.3389/fimmu.2022.1021935)

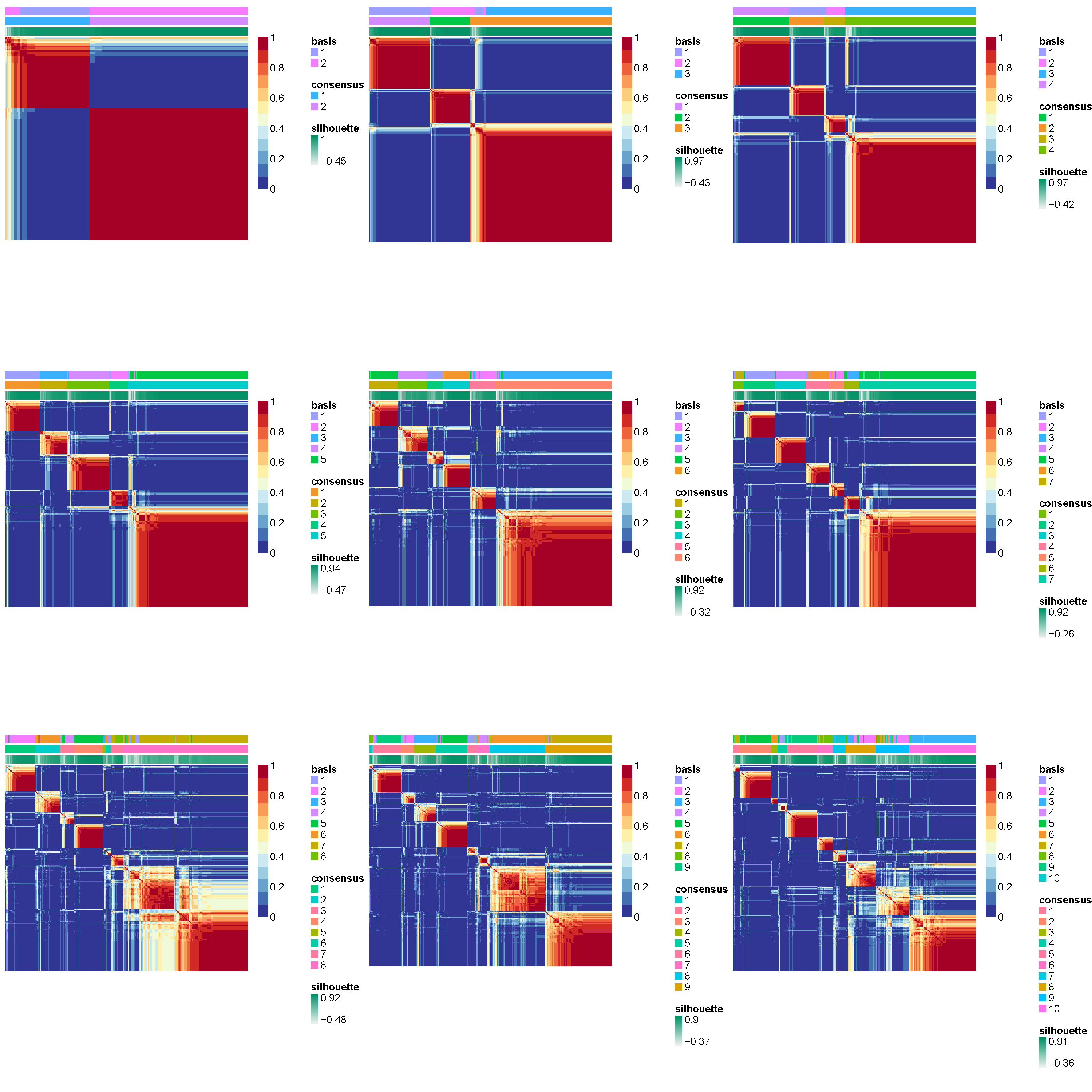

Supplement: Supplementary Figure 1 — The visual inspection of the consensus matrix by nonnegative matrix factorization. [file Image_1.tiff]

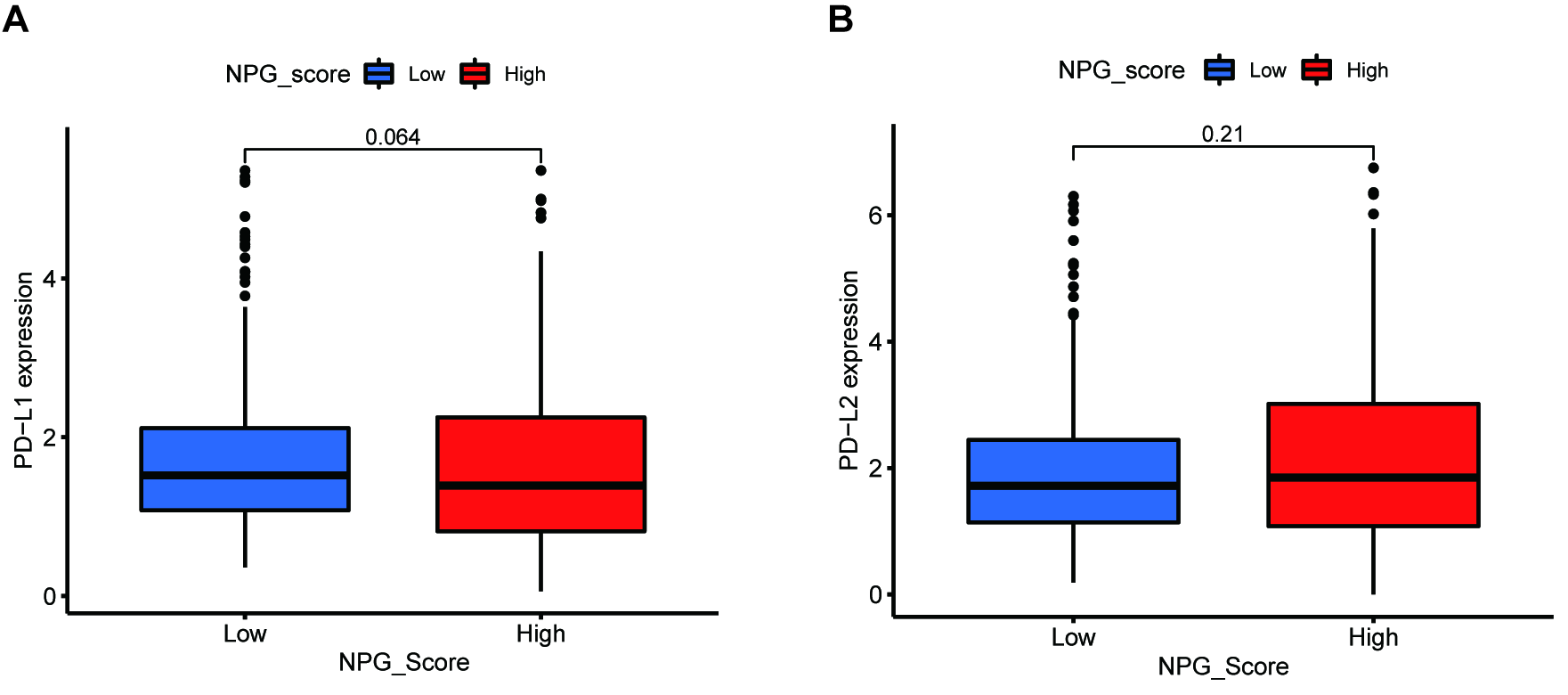

Supplement: Supplementary Figure 2 — Difference in PD-L1 and PD-L2 expression levels between high and low NPG score groups. [file Image_2.tif]

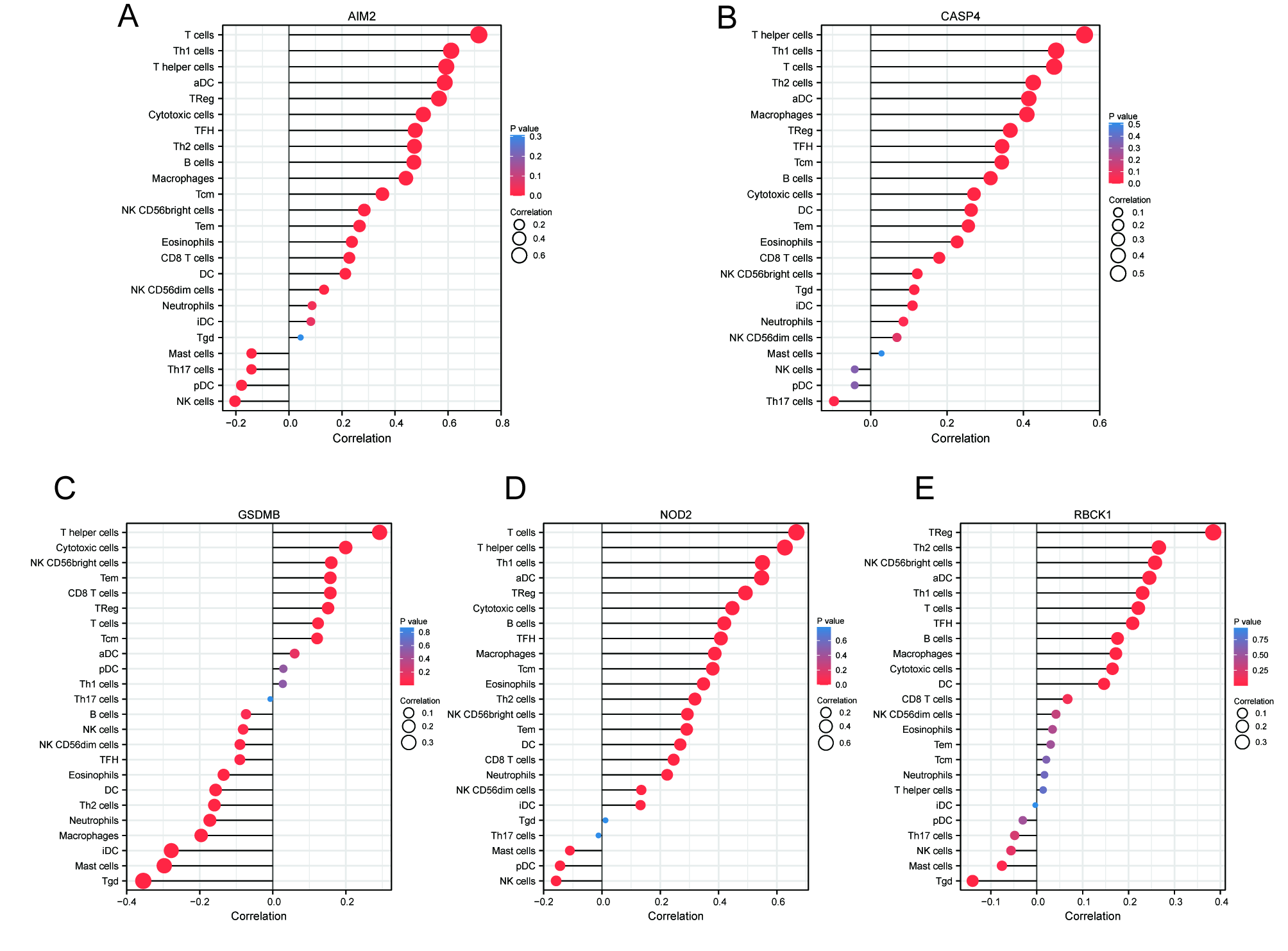

Supplement: Supplementary Figure 3 — Correlations between the abundance of immune cells and AIM2, CASP4, GSDMB, NOD2, and RBCK1. [file Image_3.tif]

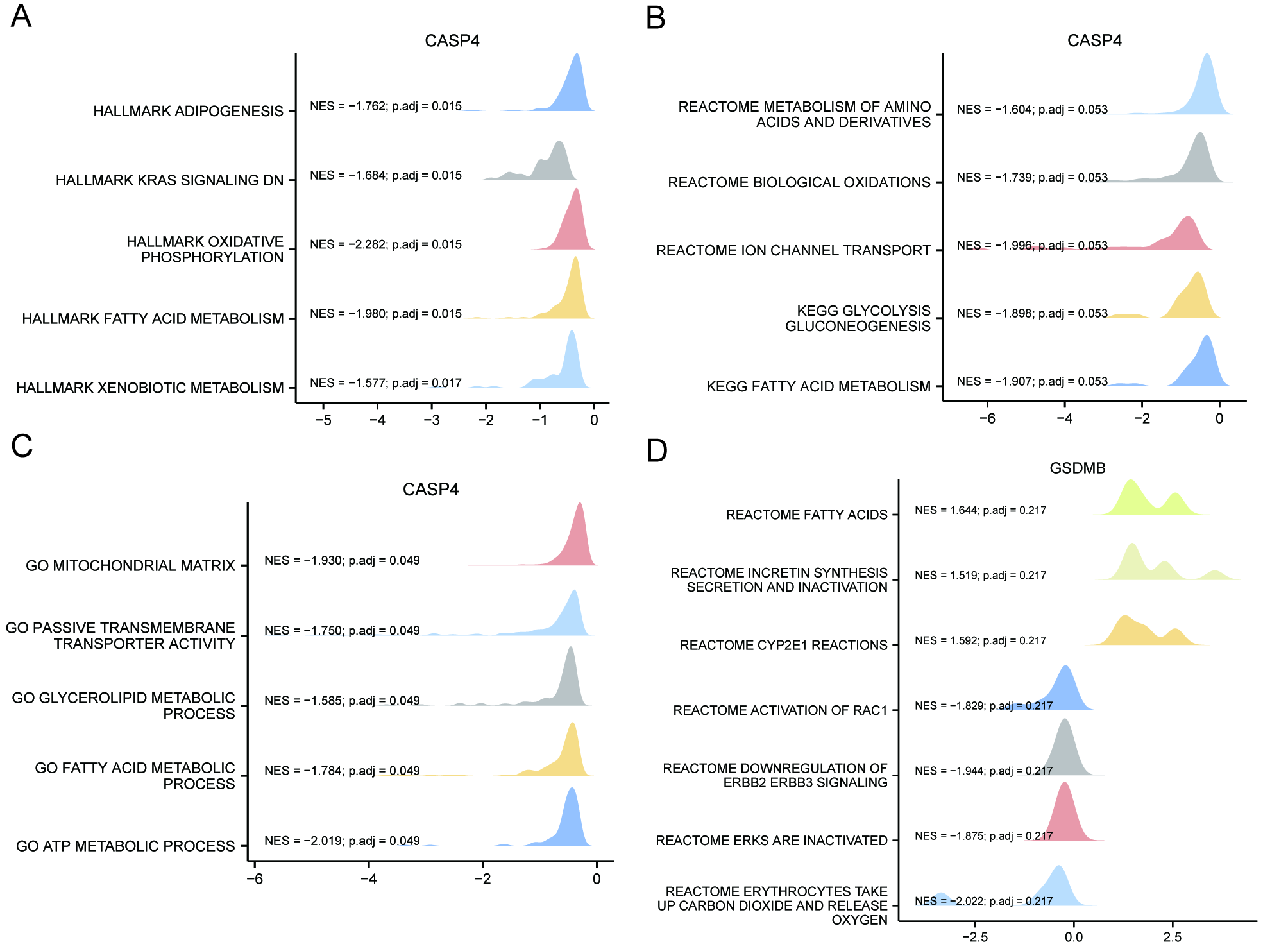

Supplement: Supplementary Figure 4 — Functional annotation of CASP4 and GSDMB by GSEA. [file Image_4.tif]
