# Supplementary material for: Crosstalk of necroptosis and pyroptosis defines tumor microenvironment characterization and predicts prognosis in clear cell renal carcinoma
Source: Front Immunol. 2022 Sep 30;13:1021935. doi: 10.3389/fimmu.2022.1021935 (PMC9561249; doi:10.3389/fimmu.2022.1021935)
Supplement: Supplementary file 6 [file Table_2.docx]

**Table S2** The clinicopathological characteristics of KIRC patients in GEO cohort

| **Characteristic** | **levels** | **Overall** |
| --- | --- | --- |
| n |  | 39 |
| Age, n (%) | <60 | 15 (38.5%) |
|  | >=60 | 24(61.5%) |
| T stage, n (%) | T1-T2 | 16 (41.0%) |
|  | T3-T4 | 23 (59.0%) |
| N stage, n (%) | N0 | 31 (79.5%) |
|  | N1 & N2 | 8 (20.5%) |
| M stage, n (%) | M0 | 25 (64.1%) |
|  | M1 | 14 (35.9%) |
| Progression, n (%) | Yes | 22 (56.4%) |
|  | No | 17 (43.6%) |
| OS event, n (%) | Alive | 22 (56.4%) |
|  | Dead | 17 (43.6%) |
| DSS event, n (%) | Alive | 23 (59.0%) |
|  | Dead | 16 (41.0%) |

Note: OS, overall survival; DSS, disease specific survival.
