# Supplementary material for: Crosstalk of necroptosis and pyroptosis defines tumor microenvironment characterization and predicts prognosis in clear cell renal carcinoma
Source: Front Immunol. 2022 Sep 30;13:1021935. doi: 10.3389/fimmu.2022.1021935 (PMC9561249; doi:10.3389/fimmu.2022.1021935)
Supplement: Supplementary file 9 [file Table_5.docx]

Table S5. The siRNA target CASP4 and GSDMB sequence

| Gene | Targeting sequences（5'-3'） | Source | Identifier |
| --- | --- | --- | --- |
| CASP4 | GCAACTGCCTCAGTCTGAA | RiboBio Co., Ltd China | stB0001526A |
| CASP4 | GCAACGTATGGCAGGACAA | RiboBio Co., Ltd China | stB0001526B |
| CASP4 | GCACAATGGGCTCTATCTT | RiboBio Co., Ltd China | stB0001526C |
| GSDMB | GGATATGATTGCCGTTAGA | RiboBio Co., Ltd China | stB0012297A |
| GSDMB | GGACAAGTGGTTAGATGAA | RiboBio Co., Ltd China | stB0012297B |
| GSDMB | CCTTGTTGATGCTGATAGA | RiboBio Co., Ltd China | stB0012297C |
| siR Negative Control |  | RiboBio Co., Ltd China | siN0000001-1 |
